# Burden of Surgical Site Infections Associated with Select Spine Operations and Involvement of *Staphylococcus aureus*

**DOI:** 10.1089/sur.2016.186

**Published:** 2017-05-01

**Authors:** Harshila Patel, Hanane Khoury, Douglas Girgenti, Sharon Welner, Holly Yu

**Affiliations:** ^1^ LASER Analytica, Montreal, Canada.; ^2^ Pfizer Inc., Pearl River, New York.; ^3^ Pfizer Inc., Collegeville, Pennsylvania.

**Keywords:** decompression, instrumentation, spinal fusion, spine operations, *Staphylococcus aureus*, surgical site infection

## Abstract

***Background:*** Spine operations may be indicated for treatment of diseases including vertebral injuries, degenerative spinal conditions, disk disease, spinal misalignments, or malformations. Surgical site infection (SSI) is a clinically important complication of spine surgery. *Staphylococcus aureus*, including methicillin-resistant *Staphylococcus aureus* (MRSA), is a leading cause of post-spinal SSIs.

***Methods:*** PubMed and applicable infectious disease conference proceedings were searched to identify relevant published studies. Overall, 343 full-text publications were screened for epidemiologic, mortality, health care resource utilization, and cost data on SSIs associated with specified spine operations.

***Results:*** Surgical site infection rates were identified in 161 studies from North America, Europe, and Asia. Pooled average SSI and *S. aureus* SSI rates for spine surgery were 1.9% (median, 3.3%; range, 0.1%–22.6%) and 1.0% (median, 2.0%; range, 0.02%–10.0%). Pooled average contribution of *S. aureus* infections to spinal SSIs was 49.3% (median, 50.0%; range, 16.7%–100%). Pooled average proportion of *S. aureus* SSIs attributable to MRSA was 37.9% (median, 42.5%; range, 0%–100%). Instrumented spinal fusion had the highest pooled average SSI rate (3.8%), followed by spinal decompression (1.8%) and spinal fusion (1.6%). The SSI-related mortality rate among spine surgical patients ranged from 1.1%–2.3% (three studies). All studies comparing SSI and control cohorts reported longer hospital stays for patients with SSIs. Pooled average SSI-associated re-admission rate occurring within 30 d from discharge ranged from 20% to 100% (four studies). Pooled average SSI-related re-operation rate was 67.1% (median, 100%; range, 33.5%–100%). According to two studies reporting direct costs, spine surgical patients incur approximately double the health care costs when they develop an SSI.

***Conclusions:*** Available published studies demonstrate a clinically important burden of SSIs related to spine operations and the substantial contribution of *S. aureus* (including MRSA). Preventive strategies aimed specifically at *S. aureus* SSIs could reduce health care costs and improve patient outcomes for spine operations.

Surgical site infections (SSIs) are potential complications occurring after surgery. Despite the availability of prophylactic antibiotics and aseptic technique, they remain a cause for concern [1,2]. Surgical site infections are the second most common health care-associated infection in the United States, representing 22% of all such infections [3]. Although SSIs are considered a preventable post-operative outcome [4], according to the published literature on spine opeations, SSI rates have been reported to range from 0.5% to 20% [2,5–9].

The leading causal agent of SSI after spine operations is *Staphylococcus aureus* [2], with several studies reporting that the pathogen was responsible for 41% to 90% of spinal SSIs [6,10–15]. The type of spine surgical procedure impacts SSI rates [8]. Instrumentation has become an integral component of spine operations for the treatment of spinal abnormalities [5]. According to one of the largest studies investigating SSI rates after spine operations, instrumentation increases the rate of post-operative infections [16] by up to 28% [8]; this is attributed to increased exposure of the wound to air, soft tissue dissection, and muscle/skin retraction [5]. This same study also reported a 33% greater rate of SSI after spinal fusion compared with procedures without fusion [8].

The repercussions of SSIs include prolonged hospitalization, increased morbidity, worse long-term patient outcomes [5], and greater direct and indirect costs [4]. The mounting pressure to manage health care resource utilization and rising health care costs has resulted in the downsizing of reimbursement for the treatment of preventable complications [3] such as SSIs.

Current evidence-based clinical guidelines established by the North American Spine Society (NASS) include the suggested use of pre-operative prophylactic antibiotics to decrease infection rates in patients undergoing spine surgery [1]. Prophylactic antibiotics are also recommended to reduce SSIs after uninstrumented lumbar spine surgery and may be considered after instrumented spine surgery [1]. However, a consensus statement issued by NASS acknowledged that despite the availability of prophylaxis, SSIs still occur after spine surgery [1]. In addition to the sub-optimal effectiveness of prophylactic antibiotics, adherence by health care professionals to the available guidelines may be an issue. A cross-sectional survey of 163 U.S. hospitals highlighted that guidelines regarding vancomycin dosing are not applied universally [17].

The objective of this study was to review the burden of SSIs among patients who have undergone selected spine operations and the contribution of *Staphylococcus aureus*. We report recent epidemiology of these specific SSIs and their associated patient outcomes, health care resource use, and costs.

## Patients and Methods

### Study design

The focus of this study was on the following spine surgical procedures: Spinal fusion with or without instrumentation and spinal decompression (including laminotomy and laminectomy). An extensive literature search within the time period August 2008 to May 2015 was performed using the PubMed database. Language was limited to English. The searches were conducted with the following primary keywords: (Spinal surgery OR spine surgery OR lumbar spine OR spine fusion OR spinal fusion OR lumbar fusion OR instrumentation OR instrumented fusion OR decompression OR laminectomy OR laminotomy) AND (post-surgical infection OR surgical site infection OR post-operative infection OR deep surgical site infection OR *Staphylococcus aureus* OR *S. aureus* OR MRSA [methicillin-resistant *Staphylococcus aureus*] OR MSSA [methicillin-sensitive *Staphylococcus aureus*] OR methicillin resistance) in combination with each of the following groups of search terms (where [TIAB] refers to the presence of the search term in the “title or abstract” and is used to focus the search):

1. (epidemiology OR epidemiological OR prevalence OR incidence)
2. (sequelae[TIAB] OR morbidity[TIAB] OR complication[TIAB] OR disability[TIAB] OR quality of life[TIAB] OR adverse event[TIAB] OR revision[TIAB] OR mortality[TIAB] OR death[TIAB])
3. (re-operation OR re-admission OR recurrence)
4. (current treatment OR clinical practice OR current practice OR clinical treatment)
5. (burden[TIAB] OR resource[TIAB] OR hospitalization[TIAB] OR hospital[TIAB])
6. (cost OR costs OR economic OR economical OR financial)*
7. (guideline OR practice guideline)*

Because of limited available studies relevant to searches 6 and 7, the timeframe for these searches was expanded five years to include the time period August 2003 to May 2015. Criteria for exclusion throughout were randomized controlled trials, case reports, commentaries, editorials, news, letters, and studies with small populations (n < 10). Interventional studies that evaluated the effects of a given antibiotic treatment specifically (e.g., intra-wound vancomycin powder) compared with an untreated control group were excluded. However, studies that used routine or standard of care antibiotic prophylaxis, which may or may not have been indicated in the study methodology, were included.

Available conference proceedings from the Infectious Diseases Society of America (IDSA), Surgical Infection Society (SIS), and Interscience Conference of Antimicrobial Agents and Chemotherapy (ICAAC) from 2011 to 2015 were searched manually for spine operations of interest and related infections.

### Data extraction and analysis

Data extracted included country, study type, year of study, duration, and population for all outcomes of interest. Some studies have more than one study cohort (i.e., total number of study cohorts used to evaluate a given outcome of interest may exceed the total number of studies). Prevalence data were categorized as SSI (percentage of procedures that developed SSIs), *S. aureus* SSI (percentage of procedures that developed *S. aureus* SSIs), and MRSA SSI rates (percentage of *S. aureus* SSIs attributable to MRSA). Not all studies evaluating prevalence data reported all outcomes of interest (i.e., number of SSIs, *S. aureus* SSIs, and MRSA infections). The type of infection (i.e., acute or chronic) was also extracted when available, according to the length of time of development after the index procedure. The mortality rate was calculated as a percentage of patients who died after developing an SSI after spine surgery. Health care resource utilization (hospitalization) was reported as length of stay (LOS). Re-admission and re-operation rates were reported as percentages of procedures that developed SSIs. Costing data were presented as the ratio between health care costs of patients undergoing spine surgery complicated by SSIs and those of patients without SSIs. The data were synthesized using descriptive statistics, including pooled averages, medians, ranges, and ratios, where appropriate.

## Results

### Search results

**FIG. 1.**
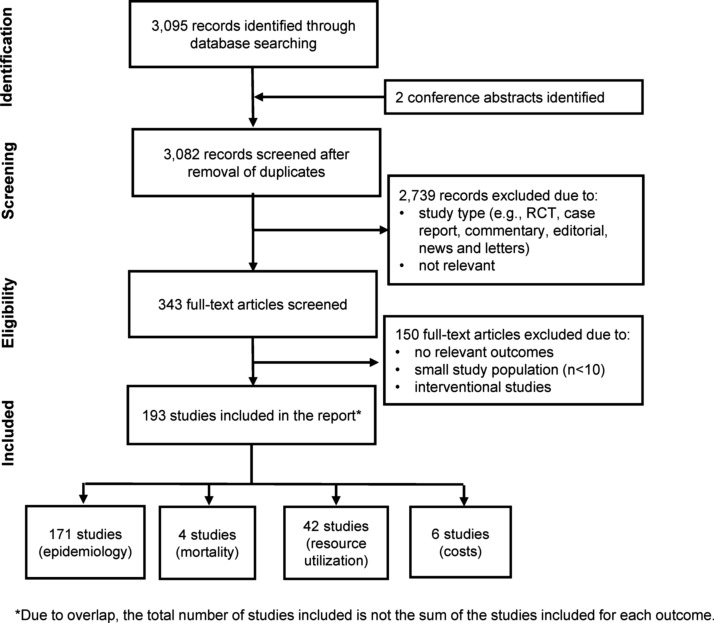
PRISMA diagram.

A total of 3,095 records were identified from the PubMed database search described previously and another two from conference abstracts (Fig. 1). After elimination of duplicates, the titles and abstracts of 3,082 records were screened according to exclusion criteria, yielding 343 references for full-text screening. A final 193 studies were deemed relevant for inclusion in this review.

### Surgical site infection rates

**FIG. 2.**
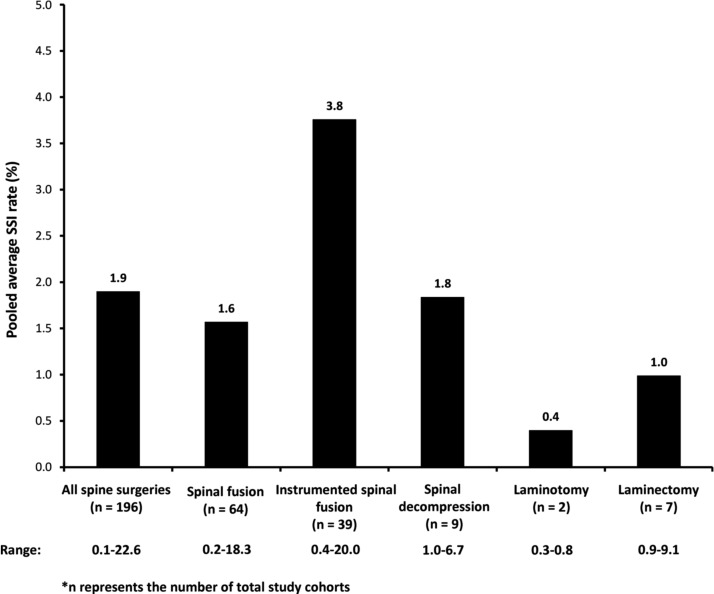
Pooled average surgical site infection (SSI) rates according to category of spine surgery.

Of the 171 available studies reporting epidemiology data, 161 evaluated SSI rates among 425,180 patients who underwent spine operations of interest (Fig. 2) [6,7,9–15,18–169]. The pooled average SSI rate was calculated to be 1.9% (median, 3.3%; range, 0.1%–22.6%) based on 196 different study cohorts (some studies had more than one cohort). Among these studies, 29 classified SSIs according to the time of onset following the index procedure [11,13,15,20,22,25,32,37,54,56,66,78,82–84,86,95,117,132,142,144,147,154,156,160,164,167,170,171]. The majority of studies used the more common Tsukayama et al. [172] classification system, in which acute (early) infections occur within one month of the index procedure and chronic (late) infections occur more than one month after the index procedure. The pooled average early SSI rate among 14,517 patients was 2.1% (median, 2.6%; range, 0.5%–16.7%) [11,13,20,22,25,32,78,82–84,86,142,147,156,164,167,170,171] compared with 0.8% (median, 0.9%; range, 0.1%–4.7%) for pooled average late SSI rate among 12,238 patients [11,13,54,83,167,171]. In terms of specific types of spine operations, 52 studies evaluated SSI rates among patients who underwent spinal fusion [9,10,14,15,21,24,27,30,33,42,43,46,52,56,58,62,63,68–70,72,75,76,92,103,105,107–109,111,113–115,119,120,123,125,128,133,139,141–144,146,150,151,157,161,162,164,173]. The pooled average SSI rate was calculated to be 1.6% (median, 2.8%; range, 0.2%–18.3%) based on 64 cohorts comprising a total of 212,639 patients. Patients who underwent instrumented spinal fusion procedures were evaluated for SSIs in 35 identified studies [6,12,25,28,35,37,39,48,50,61,67,74,77,78,84,85,90,93,97,98,101,112,121,122,126,127,132,134,135,137,148,154,156,160,174]. The pooled average SSI rate was calculated to be 3.8% (median, 4.2%; range, 0.4%–20%) based on 39 cohorts with a total of 28,628 patients. Furthermore, we identified six studies that evaluated SSI rates among patients who underwent laminectomy [10,21,65,80,82,102]. The pooled average SSI rate was calculated to be 1% (median, 2.8%; range, 0.9%–9.1%) based on seven cohorts with a total of 26,552 patients. Seven studies were identified that evaluated SSI rates among patients who underwent spinal decompression [26,40,41,66,79,124,147]. The pooled average SSI rate was calculated to be 1.8% (median, 2.4%; range, 1%–6.7%) based on nine cohorts with a total of 8,057 patients.

### *Staphylococcus aureus* rates

A total of 39 studies evaluating *S. aureus* SSI rates among patients who underwent spine operations of interest were included in this study (Table 1A) [6,7,10–15,32,37,45,47,48,52,53,62,77,84,90,91,101,103,111–113,117,122,126,129,132,137,138,140,146,155,160,164,167,173]. The pooled average *S. aureus* SSI rate was calculated to be 1% (median, 2%; range, 0.02%–10%) based on 42 cohorts evaluating a total of 112,135 patients. Among eight of these studies, which categorized *S. aureus* SSIs according to time of onset following index procedure [32,37,84,101,117,132,164,167], the pooled average early (less than one month) *S. aureus* SSI rate was 2.5% (median, 2.8%; range, 1.4%–5.4%) [32,84,164,167] among 1,017 patients compared with a single study with 737 patients reporting a late (more than one month) *S. aureus* SSI rate of 0.4% [167]. An assessment of the *S. aureus* SSI rates after specific types of spine operations was only possible for spinal fusion and instrumented spinal fusion because of availability of data. On the basis of nine studies with nine cohorts consisting of a total of 9,604 patients who underwent spinal fusion, the calculated pooled average rate was 1.8% (median, 2.6%; range, 1.1%–8.3%) [14,15,52,62,103,113,146,164,173]. According to 13 studies consisting of 14 cohorts with a total of 14,835 patients who underwent instrumented spinal fusion, the calculated pooled average rate was 1.4% (median, 2%; range, 0.1%–10%) [6,37,48,77,84,90,101,112,122,126,132,137,160]. The pooled average contribution of *S. aureus* infections to spinal SSIs was calculated to be 49.3% (median, 50%; range, 16.7%–100%; 2,272 SSIs) [6,7,10–15,32,37,45,47,48,52,53,62,77,84,90,91,101,103,111–113,117,122,126,129,132,137,138,140,146,155,160,164,167,173].

**Table 1A.** Pooled Average and Median *Staphylococcus aureus* Surgical Site Infection Rates among Patients Who Underwent Spine Operations

*Type of spine operation*	*Total number of studies (total number of cohorts)*	*Total number of patients who underwent spine operations*	*Pooled average* S. aureus *SSI rate (% of spine surgical patients)*	*Median* S. aureus *SSI rate (% of spine surgical patients)*	*Range*
All types	39 (42)	112,135	1.0	2.0	0.02–10.0
Early infection^a^	4 (5)	1,017	2.5	2.8	1.4–5.4
Late infection^a^	1 (1)	737	0.4	0.4	NA
Spinal fusion	9 (9)	9,604	1.8	2.6	1.1–8.3
Instrumented spinal fusion	13 (14)	14,835	1.4	2.0	0.1–10.0

^a^ Not all studies classified *S. aureus* infections as early or late according to the Tsukayama et al.^170^ classification system.

SSI = surgical site infection; NA = not applicable for a single study.

### Methicillin-resistant *Staphylococcus aureus* rates

There were 30 studies that assessed the proportion of *S. aureus* SSIs after spine operations of interest that were attributed to MRSA (Table 1B) [6,10–14,22,25,32,37,45,53,62,84,90,111,113,117,122,126,129,137,138,146,155,160,164,167,175,176]. The pooled average proportion of *S. aureus* SSIs attributable to MRSA was calculated to be 37.9% based on 32 cohorts with a total of 1,071 patients experiencing *S. aureus* SSIs (median, 42.5%). According to seven studies reporting early MRSA (less than one month), this proportion was slightly greater at 52.4% (median, 100%) among a total of 42 patients experiencing *S. aureus* SSIs [22,25,32,84,90,164,167]. A single study investigating involvement of MRSA in late (more than one month) SSI found that it was not present in the three patients experiencing *S. aureus* SSIs [167]. On the basis of six studies evaluating patients who underwent spinal fusion, this proportion was calculated to be 24.6% among a total of 175 patients experiencing *S. aureus* SSIs (median, 29.4%) [14,62,113,146,164,175]. Furthermore, according to 10 studies with patients who underwent instrumented spinal fusion, this proportion was calculated to be 35.5% among a total of 166 patients experiencing *S. aureus* SSIs (median, 38.8%) [6,25,37,84,90,122,126,137,160,176].

**Table 1B.** Pooled Average and Median Proportion of *Staphylococuss aureus* Surgical Site Infections Attributed to Methicillin Resistance among Patients Who Underwent Spine Operations

*Type of spine operation*	*Total number of studies (total number of cohorts)*	*Total number of patients who developed* S. aureus *SSIs after spine operations*	*Pooled average proportion of* S. aureus *SSIs attributed to methicillin resistance*	*Median proportion of* S. aureus *SSIs attributed to methicillin resistance*	*Range*
All types	30 (32)	1,071	37.9	42.5	0–100
Early infection^a^	7 (8)	42	52.4	100.0	11.8–100
Late infection^a^	1 (1)	3	0	0	NA
Spinal fusion	6 (6)	175	24.6	29.4	3.8–54.6
Instrumented spinal fusion	10 (10)	166	35.5	38.8	0–100

^a^ Not all studies classified *S. aureus* infections as early or late according to the Tsukayama et al.^170^ classification system.

SSIs = surgical site infections; NA = not applicable for a single study.

### Mortality

In severe cases, mortality is a potential complication of spinal SSIs. We identified four studies that reported SSI-related mortality data among patients who underwent spine operations of interest. A large prospective U.S. study of 24,774 veterans who had spine surgery for fusion, decompression, or instrumentation reported a 30-d mortality rate of 1.06% among patients who developed SSI compared with 0.5% among those who had no SSI [169]. In a large retrospective Japanese study of 7,178 patients who had spine surgery, the mortality rate was reported to be 2.2% among those who developed SSIs [13]. Similarly, a relatively smaller retrospective Spanish study of 481 patients who underwent posterior spinal fusion and instrumentation reported a mortality rate of 2.3% among patients who developed deep SSIs [97]. Last, a retrospective analysis of data from a Japanese nationwide administrative inpatient database reported that among 465 patients who underwent spinal fusion surgery for atlantoaxial subluxation and had rheumatoid arthritis, the in-hospital mortality rate was 6.7% among patients who developed SSIs [46]. None of the patients without rheumatoid arthritis who went on to develop SSIs died, suggesting that patients with comorbidities may have a greater risk of SSI-related complications.

### Health care resource utilization

**FIG. 3.**
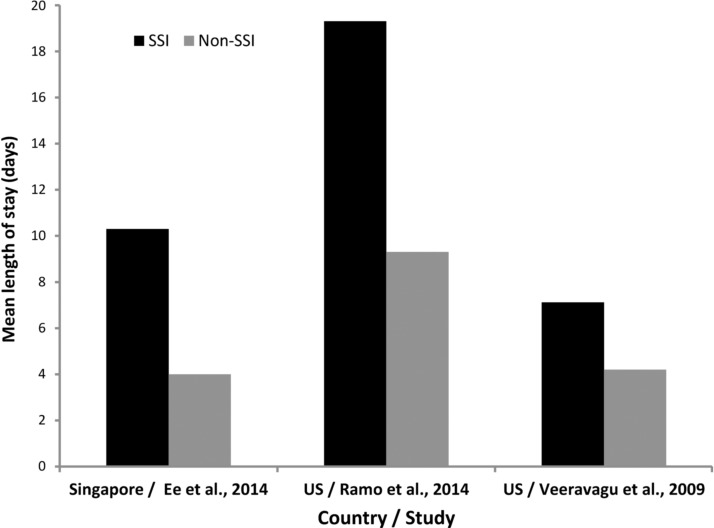
Mean length of stay for patients who underwent spine operations of interest and did or did not develop surgical site infections.

Surgical site infections are a relatively frequent source of morbidity, often requiring extended hospitalizations, prolonged antibiotic treatment, and additional surgical procedures [7]. These factors may contribute to an increased burden on health care systems. Six studies assessing hospital resource utilization as LOS by patients developing SSIs after select spine operations of interest were included in this study [11,35,53,146,169,177]. Three of these studies compared mean LOS between patients with SSIs and those without SSIs (Fig. 3); two of these studies included patients who underwent various types of spine surgical procedures [11,169], whereas the remaining included patients who underwent spinal fusion [146]. This did not make it possible to make comparisons of health care resource use attributed to SSIs across types of spine operations, but in general, patients who developed SSIs had a longer LOS (range, 7.1–19.3 d) compared with those with no SSI (range, 4.0–9.3 d), with two of the studies reporting statistical significance [11,146]. The calculated ratios of LOS among patients with SSIs versus those without ranged from 1.5 to 2.6.

**FIG. 4.**
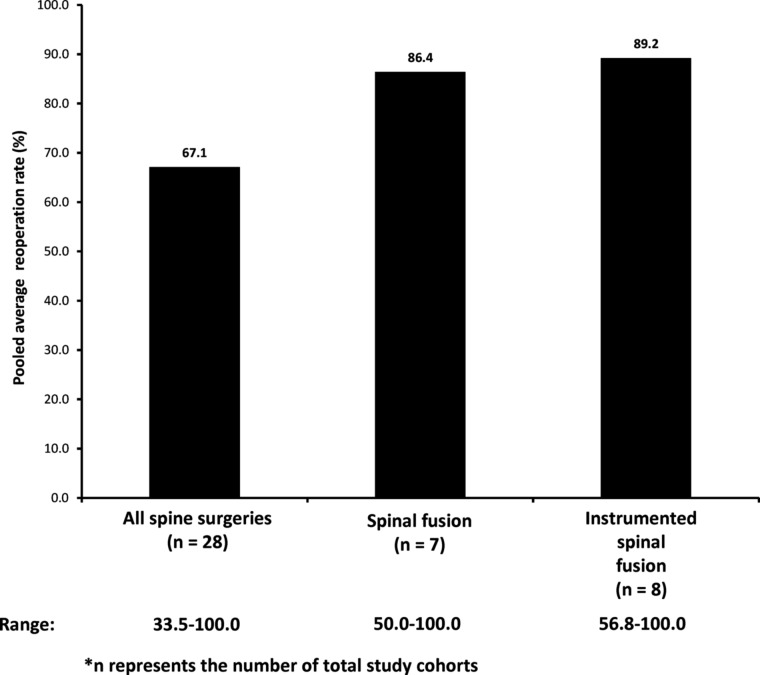
Pooled average re-operation rates caused by surgical site infection (SSI) among patients who underwent select spine operations and developed SSIs.

Unplanned hospital re-admissions, such as those caused by SSIs developing after spine operations, incur a substantial financial burden on private and public payers, hospitals, and patients themselves [124]. We identified 10 studies evaluating re-admission rates caused by SSIs developing after spine operations of interest [52,64,69,90,118,124,156,177–179]. Based on four studies with a total of 135 procedures that developed SSIs, the 30-day SSI-related re-admission rate ranged from 20% to 100% [52,69,124,156]. Re-operation rates resulting from SSIs developing after select spine operations were reported by 26 studies (Fig. 4) [11,13,20,37–39,47,48,58,72,76,78,95,97,100,106,111,112,117,121,131,132,142,147,169,173]. The pooled average re-operation rate for all identified spine surgery-related SSIs was calculated to be 67.1% (median, 100%; range, 33.5%–100%) among 1,704 procedures that developed SSIs. This rate is lower than that for instrumented spinal fusion, which was calculated to be 89.2% (median, 100%; range, 56.8%–100%) among 148 procedures that developed SSIs [37,39,48,78,97,112,121,132] and that for spinal fusion, which was calculated to be at 86.4% (median, 100%; range, 50%–100%) among 22 procedures that developed SSIs [58,72,76,111,142,173]. However, the smaller denominators should be considered when comparing the re-operation rates by type of surgery with the overall rate (i.e., 148 and 22 versus 1,704).

### Health care costs

Costs associated with SSIs resulting from spine operations of interest were reported by one Japanese [53] and five U.S. studies [9,180–183]. Only two studies reported costs related to SSIs and compared them with costs associated with non-SSI–infected patients [9,180]. Both were U.S. studies, reporting statistically significantly greater costs for patients who develop SSIs. Among patients undergoing revision instrumented lumbar fusion, the mean two-year direct costs were reported as $57,513 ± $8,253 for those with SSIs compared with $32,067 ± $6,959 for the control group (p = 0.002) [180]. Among patients who underwent sub-axial dorsal cervical spinal fusion, direct health care costs were reported to be $16,970 ± $4,375 for patients with an SSI compared with $7,658 ± $2,625 for those without an infection (p < 0.0001) [9]. Furthermore, when indirect costs were also taken into consideration and the costs adjusted for inflation to 2013 values in the published study, the total cost for the infection cohort was calculated to be $21,778 ± $5,625 for the infection cohort (versus $9,159 ± $4,087 for the non-infection cohort) [9]. Both studies demonstrate that spine surgical patients incur approximately double the health care costs when they develop an SSI.

## Discussion

There has been an exponential increase in the number of spine surgical procedures in the United States in the past two decades [70]. It may be expected that the number of post-operative complications including SSIs will also increase. However, SSIs are believed to be largely avoidable patient outcomes. The development of SSIs is perceived to reflect the quality of care provided by a given health care institution and can result in a negative grading and financial penalties [184]. Surgical site infections have become the target of cost reduction measures by an increasingly burdened health care system [70]. The objective of this review was to identify and characterize the SSI rate among patients undergoing spine operations of interest, describe the contribution of *S. aureus*, and evaluate the resulting clinical and economic impact.

Based on 161 studies included, the pooled average SSI rate among spine operations of interest was calculated to be 1.9%, which is within the somewhat wide range reported in the literature [5]. When assessed by type of spine surgery, SSI rate for instrumented spinal fusion was higher than for spinal fusion and spinal decompression. This coincides with previously published reports that suggest that the higher SSI rate associated with this type of spine surgical procedure is partly attributed to its greater complexity (e.g., longer duration of surgical procedure, increased need for instrumentation, retractor usage, and soft tissue dissection) [5]. Furthermore, when evaluating the time of onset of SSI after the index spine surgical procedure, the higher pooled average rate for early versus late infections (2.1% versus 0.8%) suggests that the first 30 d are the most crucial period for acquiring SSIs. The pooled average *S. aureus* SSI rate was calculated to be 1%, which is a little more than half the pooled average SSI rate in this study. Furthermore, the pooled average contribution of *S. aureus* infections to spinal SSIs was calculated to be 49.3%. This agrees with *S. aureus* reported as being the major pathogen responsible for SSIs.

The SSI rates among spine surgical patients are not negligible, as supported by this study. Furthermore, when they do develop, their management is challenging and frequently require additional health care resources [7] to prevent detrimental sequelae (e.g., acute neurologic decompensation, epidural abscess, death) [9]. In this review, the SSI-related mortality rates among patients who underwent spine surgery ranged from 1.06% to 2.3% based on three studies. Thus, treatment for SSI needs to be aggressive and often necessitates surgical debridement and antibiotic therapy [9]. In our review, there were too few studies on a given type of spine surgical procedure to make comparisons, however, the development of SSIs resulted consistently in noticeably longer LOS. In situations in which SSIs develop after discharge, patients frequently need to be re-admitted if they require a medical intervention. The 30-d re-admission rate in this study ranged from 0.5% to 4.8%. Surgical site infection-related re-admissions, including among patients who underwent spine surgery, are yet another source of costly burden on the health care system [124]. They are considered a key undesirable outcome by the World Health Organization [185] and are the major target for cost reduction measures via mandates of the Patient Protection and Accountable Care Act of 2010 [124]. In the event that a further surgical intervention is required upon re-admission, spine surgical patients with SSIs will additionally impact the re-operation rate. The pooled average SSI-related re-operation rate for spine operations of interest was calculated to be 67.1% in this study.

Limitations to this study should be noted. The majority of studies identified were from North America (predominantly the United States), Europe, and Asia. The paucity of data reporting SSI rates and their associated complications among spine surgical patients from other geographic regions including South America and Africa highlights an important gap in the published literature in this field. Incomplete or unclear study methodologies often prevented a more in-depth analysis (e.g., standard error) of SSI rates, necessitating comparisons that were restricted to crude analysis (e.g., pooled averages and ratios). It is also noteworthy that not all studies evaluating prevalence data reported all the outcomes of interest for this literature review (e.g., a study reporting *S. aureus* SSIs may not necessarily report total SSIs). Similarly, not all studies classified SSIs as late or early according to the definition used in this study. This explains why there were more studies reporting early infections among MRSA infections (n = 9) than among *S. aureus* infections (n = 4). In the case of health care resource utilization, different outcome measures were often reported for hospitalizations and the most common outcome (i.e., LOS) was chosen to make meaningful comparison across studies that included a control group with no SSIs. Direct comparisons of costs were not feasible due to differences in years of costing and currency. Furthermore, the absence of definitions for acute (early) and chronic (late) SSIs as time of onset after index surgical procedure restricted comparisons across studies that used a common definition.

Another key limitation is the inconsistency in the reporting of the use of standard of care, which usually consists of pre-operative systemic antibiotic prophylaxis. Several studies did not state specifically its use in their study population; because it has been reported that the administration of pre- and post-operative prophylactic antibiotics is not always recorded by institutions [8] and that its application can vary [160], a possible explanation is provided for the large range of SSI rates reported across studies in this review.

The persistence of SSIs after spine operations despite the availability of prophylactic antibiotics [1] highlights the need for an alternate strategy that focuses on prevention. Furthermore, specifically targeting the more common pathogen, *S. aureus*, may reduce avoidable SSI-related health care costs and improve patient outcomes.
